# Ligation of Left Renal Vein for Spontaneous Splenorenal Shunt to Prevent Portal Hypoperfusion after Orthotopic Liver Transplantation

**DOI:** 10.1155/2013/842538

**Published:** 2013-02-28

**Authors:** Lampros Kousoulas, Kristina Imeen Ringe, Michael Winkler, Frank Lehner, Nicolas Richter, Juergen Klempnauer, Fabian Helfritz

**Affiliations:** ^1^Department of General, Visceral and Transplant Surgery, Hannover Medical School, Carl-Neuberg-Street 1, 30625 Hanover, Germany; ^2^Institute of Radiology, Hannover Medical School, Carl-Neuberg-Street 1, 30625 Hanover, Germany

## Abstract

We report a case of recovered portal flow by ligation of the left renal vein on the first postoperative day after orthotopic liver transplantation of a 54-year-old female with alcoholic liver cirrhosis, chronic kidney failure, and spontaneous splenorenal shunt. After reperfusion, Doppler ultrasonography showed almost total diversion of the portal flow into the existing splenorenal shunt, but because of severe coagulopathy and diffuse bleeding, ligation of the shunt was not attempted. A programmed relaparotomy was performed on the first postoperative day, and the left renal vein was ligated just to the left of the inferior vena cava. Portal flows subsequently increased to 37 cm/sec, and the patient presented a good and stable liver function. We conclude that patients with known preoperative splenorenal shunts should be closely monitored, and if the portal flow becomes insufficient, ligation of the left renal vein should be attempted in order to optimize the portal perfusion of the liver.

## 1. Introduction

In cirrhotic patients with portal hypertension, collateral vessels into systemic circulation are well known. The amount of collateral flow depends on the stage of portal hypertension. In advanced stages, the development of a reversal hepatofugal portal flow may lead to a portal steal syndrome [1]. After orthotopic liver transplantation, usually the portal flow and pressure normalize and, providing that there is an adequate-sized graft, collateral vessels collapse and obliterate [2–4].

Low portal vein flows after orthotopic liver transplantation, due to persisting splenorenal shunt, are associated with hepatic hypoperfusion and poor allograft survival [2]. Splenorenal shunts are present in cirrhotic patients from nearly 14% up to 21%, and several studies have suggested that spontaneous portosystemic shunts should be treated in order to recover the portal flow of the liver graft [5, 6]. Beside direct division of the shunt vessels with or without splenectomy, the ligation of the left renal vein is described to be an effective technique and has been reported to be safe in adult liver transplant patients with large splenorenal shunts [7–9].

## 2. Case Report

The patient is a 54-year-old female with alcoholic liver cirrhosis and chronic kidney failure, listed for liver and sequential renal transplantation. The patient underwent a percutaneous ethanol injection therapy for a solitary hepatocellular carcinoma in 2009. At the timepoint of transplantation the MELD score was 37.

**Figure 1:**
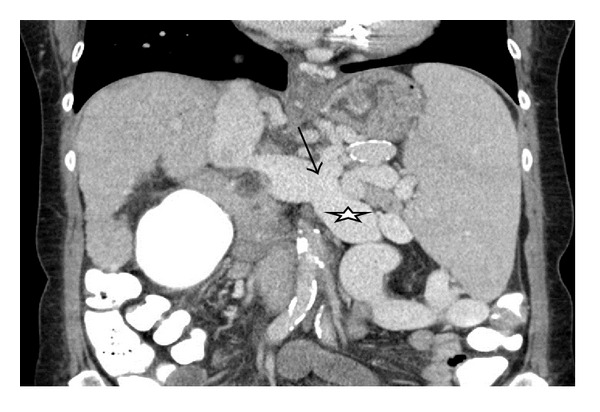
Preoperative CT imaging showing the splenorenal shunt (arrow) and a splenomegaly (left renal vein = star).

The preoperatively conducted abdominal computed tomographic (CT) scan showed severe portal-systemic collateral vessels of the abdomen, including a splenorenal shunt (Figure 1).

The patient underwent an orthotopic liver transplantation using a full-size organ. Donor age was 56 years, and the organ quality was rated as “acceptable” by the explant surgeon. Histopathological rating of steatosis was 25–30%. The center standardised transplant procedure was performed with replacement of the retrohepatic inferior vena cava and without any bypass procedure. Anastomosis time was 43 min, incision to suture time was 3 hours 09 min, and cold ischemic time was 8 hours 47 min.

**Figure 2:**
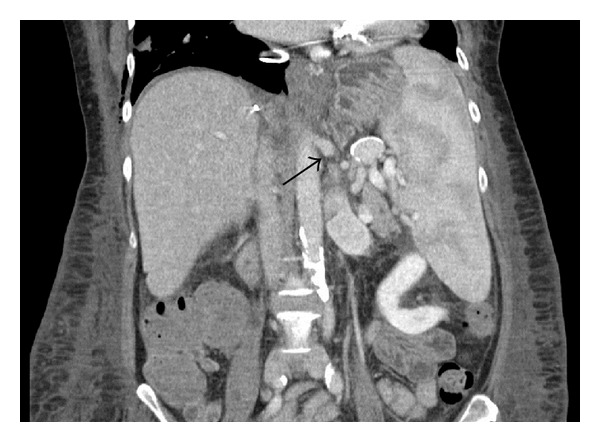
Postoperative CT imaging (day 5) after the ligation of the left renal vein. The arrow shows the point of ligation.

After reperfusion, Doppler ultrasonography showed total diversion of the portal flow into the existing splenorenal shunt, but because of severe coagulopathy and diffuse bleeding, ligation of the shunt was not attempted. A programmed relaparotomy was performed on the first postoperative day, and the left renal vein was ligated at its confluence to the inferior vena cava (Figure 2).

Portal flows subsequently increased to 37 cm/sec. The postoperative graft function was excellent and substitution of plasma or coagulation factors was not necessary. The postoperative Doppler ultrasound examination showed normal flows for both the hepatic artery and portal vein. The further postoperative course was uncomplicated.

**Figure 3:**
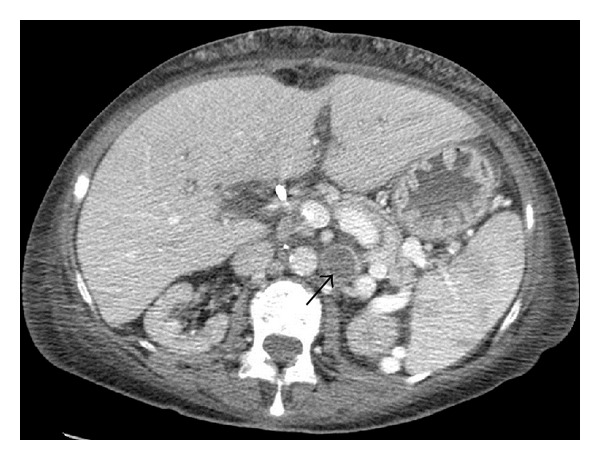
Postoperative CT imaging (day 47) showing the thrombosis of the left renal vein at the point of ligation (arrow).

**Figure 4:**
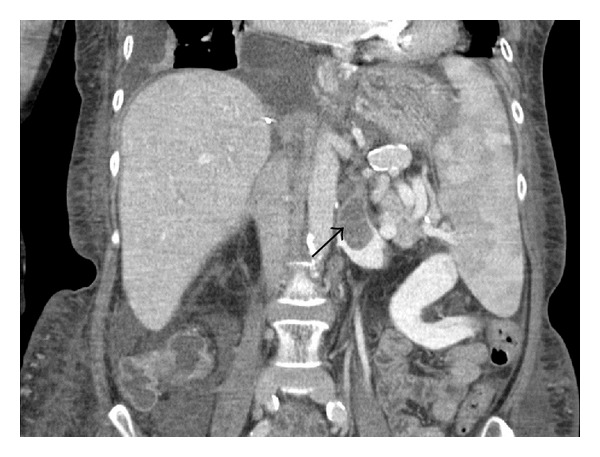
Postoperative CT imaging (day 47) showing in axial form the thrombosis of the left renal vein (arrow).

About two months after the liver transplantation, another CT scan of the abdomen was performed, and progredient thrombosis of the left renal vein was observed (Figures 3 and 4). Due to the preexisting chronic renal failure, this fact was without any consequence for our patient, but it demonstrates that the procedure of renal vein ligation bears the potential risk of renal impairment. The patient currently enjoys good allograft function with normal liver function tests.

## 3. Discussion

Preexisting splenorenal shunts may lead to insufficient portal flow after orthotopic liver transplantation, which is associated with hepatic hypoperfusion and poor allograft survival [10, 11]. As an adequate portal venous inflow is critical for graft function and survival, spontaneous or surgically created portosystemic shunts have to be treated after the liver transplantation in order to improve the portal flow to the allograft [12]. Direct ligation of a splenorenal shunt, with or without splenectomy, is technically challenging and is associated with a high risk of bleeding or of infection [13, 14]. Furthermore, the ligation of the shunt may not always significantly improve the portal flow. Therefore, the ligation of the left renal vein at the inferior vena cava has been proposed as an alternative therapeutic approach. Given the fact that the venous renal blood flow accelerates the portal flow after the ligation of the renal vein, this procedure can lead to sufficient portal flow.

Our case report shows that the ligation of the left renal vein can be performed safely in order to optimize the flow of the portal vein after the liver transplantation, but this procedure could also lead to renal impairment due to outflow problems and thrombosis of the left renal vein [2, 7, 8]. In our case, because of the chronic kidney failure of the patient, the decision to ligate the left renal vein was easily made, but in patients with normal kidney function, this aspect should be taken into consideration.

Thus, we conclude that patients with known preoperative splenorenal shunt should be closely monitored after orthotopic liver transplantation, and if the portal flow becomes insufficient, direct ligation of the splenorenal shunt or ligation of the left renal vein at the inferior vena cava should be attempted in order to prevent portal flow steal and optimize the portal perfusion of the liver. The selection of the method performed should be based not only on the ability to identify and ligate the shunt vessels, but also on the special characteristics of the patient and on the experience of the surgeon.

## References

[1] von Herbay A, Frieling T, Haussinger D (2000). Color Doppler sonographic evaluation of spontaneous portosystemic shunts and inversion of portal venous flow in patients with cirrhosis. *Journal of Clinical Ultrasound*.

[2] Castillo-Suescun F, Oniscu GC, Hidalgo E (2011). Hemodynamic consequences of spontaneous splenorenal shunts in deceased donor liver transplantation. *Liver Transplantation*.

[3] Kita Y, Harihara Y, Sano K (2001). Reversible hepatofugal portal flow after liver transplantation using a small-for-size graft from a living donor. *Transplant International*.

[4] De Carlis L, Del Favero E, Rondinara G (1992). The role of spontaneous portosystemic shunts in the course of orthotopic liver transplantation. *Transplant International*.

[5] Tarantino G, Citro V, Conca P (2009). What are the implications of the spontaneous spleno-renal shunts in liver cirrhosis?. *BMC Gastroenterology*.

[6] Zardi EM, Uwechie V, Caccavo D (2009). Portosystemic shunts in a large cohort of patients with liver cirrhosis: detection rate and clinical relevance. *Journal of Gastroenterology*.

[7] Lee SG, Moon DB, Ahn CS (2007). Ligation of left renal vein for large spontaneous splenorenal shunt to prevent portal flow steal in adult living donor liver transplantation. *Transplant International*.

[8] Slater RR, Jabbour N, Abbass AA (2011). Left renal vein ligation: a technique to mitigate low portal flow from splenic vein siphon during liver transplantation. *American Journal of Transplantation*.

[9] Cho SY, Kim SH, Lee KW, Park SJ, Han SS, Kim YK (2009). Ligation of left renal vein as a salvage procedure for splenorenal shunt after living donor liver transplantation: a case report. *Transplantation Proceedings*.

[10] Sadamori H, Yagi T, Matsukawa H (2008). The outcome of living donor liver transplantation with prior spontaneous large portasystemic shunts. *Transplant International*.

[11] Shapiro RS, Varma CVR, Schwartz ME, Miller CM (1997). Splenorenal shunt closure after liver transplantation: intraoperative Doppler assessment of portal hemodynamics. *Liver Transplantation and Surgery*.

[12] Sadamori H, Yagi T, Shinoura S New surgical approach to large splenorenal shunt in living donor liver transplantation: diversion of SMV and SPV blood flow. *Journal of Gastrointestinal Surgery*.

[13] Settmacher U, Nüssler NC, Glanemann M (2000). Venous complications after orthotopic liver transplantation. *Clinical Transplantation*.

[14] Troisi R, Hesse UJ, Decruyenaere J (1999). Functional, life-threatening disorders and splenectomy following liver transplantation. *Clinical Transplantation*.

